# Diagnosis and Treatment Approaches in Infantile Colic (IC): Results of a Survey Among Paediatricians in Turkey

**DOI:** 10.3389/fped.2021.779997

**Published:** 2021-12-23

**Authors:** Samil Hizli, Demet Can, Ilknur Kiliç, Emel Örün, Turan Tunç, Hasan Özkan

**Affiliations:** ^1^Division of Pediatric Gastroenterology, Hepatology and Nutrition, Ankara Yildirim Beyazit University, Ankara, Turkey; ^2^Division of Pediatric Allergy and Immunology, Balikesir University, Balikesir, Turkey; ^3^Division of Neonatology, Ataşehir Florence Nightingale Hospital, Istanbul, Turkey; ^4^Division of Social Pediatrics, Liv Hospital, Ankara, Turkey; ^5^Division of Neonatology, Ataşehir Memorial Hospital, Istanbul, Turkey; ^6^Division of Neonatology, Dokuz Eylül University, İzmir, Turkey

**Keywords:** infantile colic, infantile colic management, simethicone, probiotics, Turkey

## Abstract

**Background and Objective:** Due to limited knowledge on the etiopathogenesis of infantile colic (IC) and the insufficiency of data regarding current treatments, different approaches emerge in terms of diagnosis, and treatment modalities globally and also in Turkey. The objective of this study was to observe how infantile colic is diagnosed and treated by paediatricians in Turkey.

**Methods:** An anonymous electronic questionnaire was used to collect the respondents' opinions. The study questionnaire was comprised of 4 different sections with 56 multiple-choice questions covering demographic features, diagnostic approach, treatment preferences and response to treatment.

**Results:** A total of 375 paediatricians responded to the survey. Fifty three percent of the participants stated that they established the IC diagnosis based only on their clinical experience. Factors that most affected the decision to start treatment were identified as parent discomfort, decreased family quality of life, and crying duration (68, 66, and 54%, respectively). Application of soothing methods, probiotics, and simethicone were identified as the most frequently used treatment modalities (frequency ranking; 81, 76, and 50%, respectively). Of the participants, 98% stated that they used probiotic as supplements, on the other hand, 72% of the participants indicated that they used simethicone as the only medical treatment to treat IC. The question about the participants' observations regarding the response to probiotic treatment was answered by 71% of the participants with decreased crying duration, while easier stool/gas passage and resolved digestion problems were the other frequent observations (54 and 49%, respectively). The observations related to the response to simethicone treatment also included decreased crying duration in addition to decreased crying periods after feeding and easier gas/stool passage (67, 47, and 44%, respectively).

**Conclusions:** Survey results revealed that the majority of the paediatricians used their clinical experience alone to establish the diagnosis of IC and preferred probiotic supplements and simethicone as the only medical treatment to treat IC and they observed clinical benefits from them. Insights generated by this study will be helpful to guide future efforts to improve the management of infantile colic by paediatricians.

## Introduction

For babies, crying offers a means for communication with the environment. Normal crying patterns are defined as 2 h per day in a 2-week-old infant, 3 h in a 6-week-old infant, and 1 h in a 3-month-old infant. In most instances, crying and restlessness gradually disappear following the 3rd and 4th months of life (1). In a meta-analysis included 28 diary studies with 8,690 infants, Wolke et al. demonstrated that the mean fuss/cry duration across studies was stable at 117–133 mins (SDs: 66–70) in the first 6 weeks and dropped to a mean of 68 mins (SD: 46.2) by 10–12 weeks of age (2). Although crying is a behaviour mode of communication for infants, prolonged crying and restlessness may affect their sleep pattern, mother-infant bonding and also represent a source of anxiety for both family members and physicians (3). Moreover, numerous studies reported that, persistent infant crying associated with sleeping or feeding problems at 3 to 6 months of age have been reported to be predictors/ precursors of attention deficit/ hyperactivity and lower academic achievement during preschool and school years (4).

Excessive crying started drawing the attention of medical community in 1890s and infantile colic (IC) (the term used for this condition in clinical practise in Turkish language is “infantil kolik,” therefore, infantile colic term was preferred in this survey study and manuscript content) was first described by Wessel et al. (5). According to the definition also known as Wessel's rule or criteria of threes, IC is defined as excessive episodic crying that starts in the first weeks of life and lasts for a minimum of 3 weeks without an obvious cause, lasting for 3 h per day, and occurring on 3 or more days per week, mostly in the afternoon or evening hours. Other features of IC described by Wessel and colleagues include certain movements such as clenching of the fists, flushing, drawing up and relaxing of the legs, tense abdomen, tight closing and opening eyes, and wrinkled forehead (5). Since retrospective evaluation of crying and restlessness periods by the parents has been found to be associated with certain challenges and diagnostic errors, the Wessel criteria were considered unfeasible for practical use. Therefore, the modified Wessel's criteria of crying and/or fussing ≥3 h/day for ≥3 days/week for 1 week was started to be used for its practicality and feasibility in clinical research (6, 7). Consequently, Rome criteria, which were developed for functional gastrointestinal disorders were introduced for defining infantile colic (8, 9). According to Rome criteria, the hourly timing of crying episodes has no role in defining IC. While Rome III criteria require the absence of growth retardation in babies up to 4 months of age in addition to requiring the presence of crying patterns similar to those described by Wessel, in Rome IV the modified Wessel's criteria used in Rome III were abandoned. According to the revision committee, these criteria were arbitrary, culturally dependent, impractical, and did not reflect the impact of the child's symptoms on the family. Therefore, the new clinical criteria are based on symptoms that have been shown to cause higher distress to parents. Besides this, the age of diagnosis of infant colic was extended to infants up to 5 months of age in Rome IV criteria (10). For research purposes additional criteria were also defined to diagnose infant colic: parents have to report that their infant has cried or fussed for 3 or more hours per day, during 3 or more days in the preceding week. In addition, parents have to keep a 24-h behaviour diary to confirm that the total amount of crying and fussing is more than 3 h per 24 h (10).

IC is defined as one of the most common presentations to the primary healthcare providers in early life and efforts at relieving IC symptoms are known to be associated with significant economic impact. For instance, in a 1997 study from the UK, the annual cost of treatments in infants up to 3 months of age experiencing persistent crying and sleep problems was estimated to be 65 million pounds (11). IC studies using different IC criteria have reported global IC prevalence ranging between 3 and 40% (12). In Wolke et al.'s meta-analysis study, colic was much more frequent in the first 6 weeks (17–25%) compared with 11% by 8–9 weeks of age and 0.6% by 10–12 weeks of age, according to modified Wessel criteria (2). There is limited data regarding the prevalence and incidence of IC in Turkey. In a study from the eastern part of Turkey, prevalence of infantile colic was reported as 19.9 % among infants between 3 weeks-3 months of age (13).

Various surveys have been conducted to examine the diagnostic and therapeutic management strategies of physicians in IC and to investigate the nutritional habits and the level of anxiety, restlessness and tension among parents resulting from their babies (14–16). To date, no such surveys regarding infantile colic have been conducted with physicians in Turkey. The present questionnaire-based study of infantile colic performed among a group of Turkish paediatricians aimed to assess the diagnostic methods, therapeutic choices and treatment management, and their observed benefits.

## Methods

### Development of the Questionnaire

Due to the lack of a validated assessment tool in IC, a systematic literature review was performed by the researchers paediatric gastroenterologist (1), social paediatrician (1), paediatric allergy & immunologist (1), neonatologist (3) in their corresponding areas of interest, and a pool of multiple-choice questions was created, by taking into account their expert opinions. The final questionnaire included a total of 56 questions based on expert consensus achieved by unanimity. The questionnaire items collected information on the following: physicians' qualifications other than identity; the city and hospital of practise; average daily and weekly number of patients who could be potential candidates for a diagnosis of infantile colic (8); physician's approach to symptoms and signs in patients with suspicion of IC (6); treatments administered following a diagnosis, if any; therapeutic choices and treatment frequency based on infant age and nutrition pattern; parameters considered important in therapeutic decisions (12); perceptions of response to treatment (6); and in-depth information on the approach to certain treatment methods (spasmolytics, probiotics, simethicone) (24) (Supplementary Material).

### Survey Tool

The questionnaire was introduced into the Survey Monkey electronic survey system with a text explaining the purpose of the study. The electronic link for the questionnaire was sent by the investigating physicians to paediatricians via e-mails and social networks, with a notice of 2-week response time. The responses to the questionnaire were stored at SurveyMonkey.com in an encrypted electronic data format. Survey Monkey does not collect data on names, e-mail addresses, or IP addresses; therefore, the responses maintained their anonymity, and the identity of the participants remained unknown, even to the investigators. Survey Monkey application generates summary statistics and charts, with numbers rounded to the nearest whole digit.

## Results

A total of 375 paediatricians answered the questionnaire between 14th April and 5th May 2021. Each question was answered by a varying number of paediatricians.

### The Characteristics of Participating Physicians

Most participants were female physicians (61.7 vs. 38.2%), and most (87.6%) had no paediatrics subspecialty. The rate of response did not differ between paediatricians from the three biggest cities and those from other cities with a population of <2 million citizens (42.6 vs. 45.2%). The number of respondent paediatricians employed in private or state hospitals (37.6 vs. 35.8%) and those employed in research/training hospitals or academic centres (12.9 vs. 10.2%) were also comparable. Therefore, a nearly homogeneous distribution was observed among participant clinicians with respect to the type of healthcare unit in which they were employed and the place of residency.

The daily number of patients under 6 months of age seen by paediatricians was mostly between 5 and 10 (45.3%), followed by between 11 and 20 in 23.7% and <5 in 23.1% across the physicians who answered the questions. Of the participants, 42.9% reported that the average number of patients under 6 months of age whom they diagnosed with infantile colic was 1–4 (Table 1).

**Table 1:** Demographic characteristics of survey participants.

**Participant characteristic**	**Proportion to overall respondents**
**Paediatrician** (*n* = 375)^*^	
**Subspecialty**	**%** ^*^
None	87.65
Yes	12.35
**City of residency**	
The biggest 3 cities^**^	42.60
The biggest 4th to 9th cities^***^	12.17
Other cities	45.23
**Gender**	
Female	61.73
Male	38.27
**Years employed as a specialist**	
1–3 years	26.85
4–6 years	15.43
7–10 years	8.95
10 years and more	48.77
**Type of healthcare unit** ^**+**^	
State Hospital	35.80
Training and Research Hospital	12.96
University Hospital	10.19
City Hospital	3.09
Private Hospital	37.65
Doctor's Office	7.41
**Mean daily number of patients <6 months of age seen by paediatricians**	
Less than 5	23.15
5–10	45.37
11–20	23.77
21–40	6.17
More than 40	1.54
**Mean weekly number of patients <6 months of age diagnosed with IC**	
1–4	42.90
5–10	32.72
11–15	15.12
16–20	4.94
>20	4.32

* *Total number of participants = 375; number of participants responding to the section on demographics = 324; %, percentages of respondents for the specified questions were calculated in the respondents' universe*.

** *above 4 million,*

*** *above 2 million,*

+ *Certain academicians in Turkey have permission to work in both state and private sector, distribution percentages exceeding 100% are due to selecting two responses at the same time by relevant participants*.

### Diagnostic Approaches in Infantile Colic

**Figure 1:**
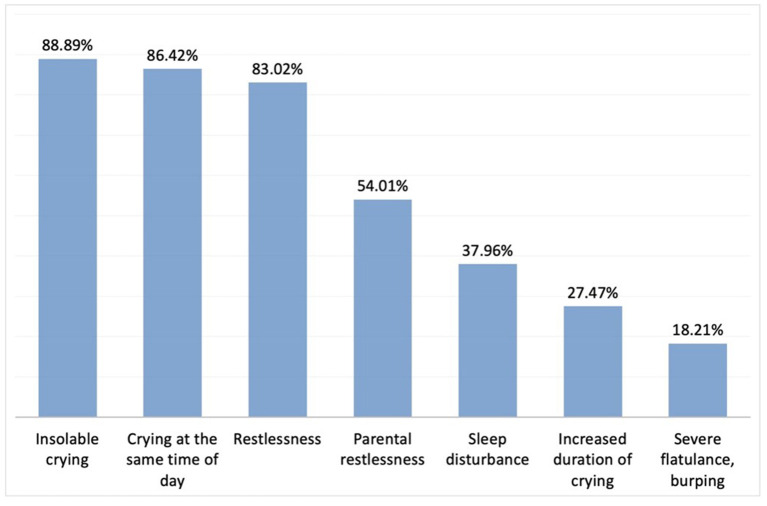
Signs and symptoms suggesting a diagnosis of IC. Here we provided the rest of the percentages of the chosen other answers: Vomiting after feeding; 5.25%, Tendency to stop receiving breast milk; 4.32%, Change in consistency (runny/watery) and odour of stool; 3.40%, Mucus in stool; 3.40%, Eczema; 1.23%, Inability to gain weight, retardation in growth percentile; 0.93% respectively.

**Figure 2:**
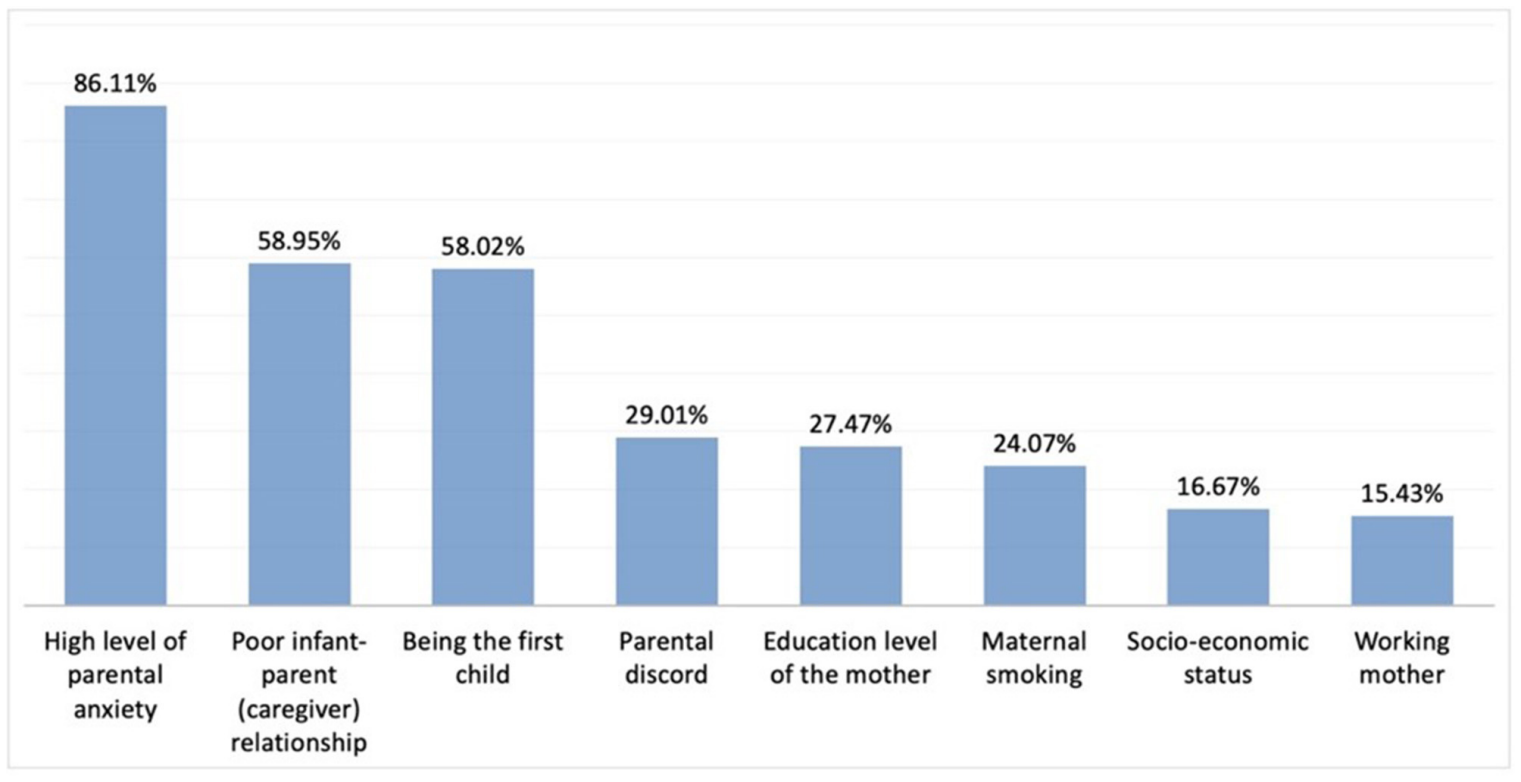
Familial factors affecting IC. Here we provided the rest of the percentages of the chosen other answers: Being a nuclear family; 11.73%, Advanced maternal age; 7.72%, None; 4.94% respectively.

Again, the questions regarding diagnostic approaches in IC were answered by 324 physicians. The most common age at which IC was diagnosed was reported to be 4–6 weeks by 49% of the participants, and 2–4 weeks by 37.6%. The 5 most common symptoms suggestive of a diagnosis of IC included inconsolable crying, crying at the same time of the day, restlessness, parental restlessness, and sleep disturbance (Figure 1). Among the familial factors associated with IC, high levels of parental anxiety was chosen as the most associated factor and advanced maternal age was chosen as the least associated factor (Figure 2).

**Figure 3:**
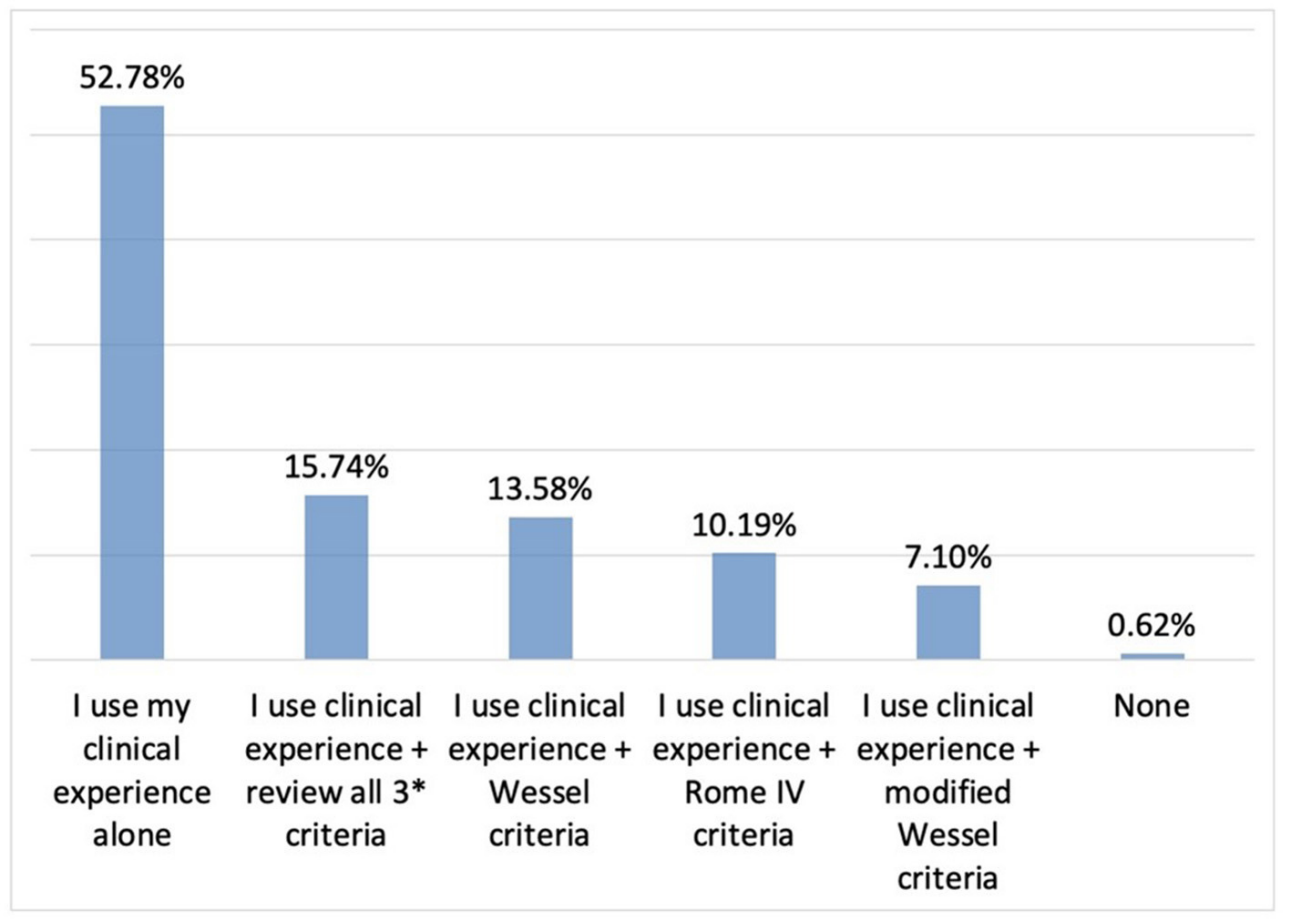
The role of clinical experience vs. diagnostic criteria in the diagnosis of IC. *3 criteria: Wessel, Rome IV, modified Wessel.

**Figure 4:**
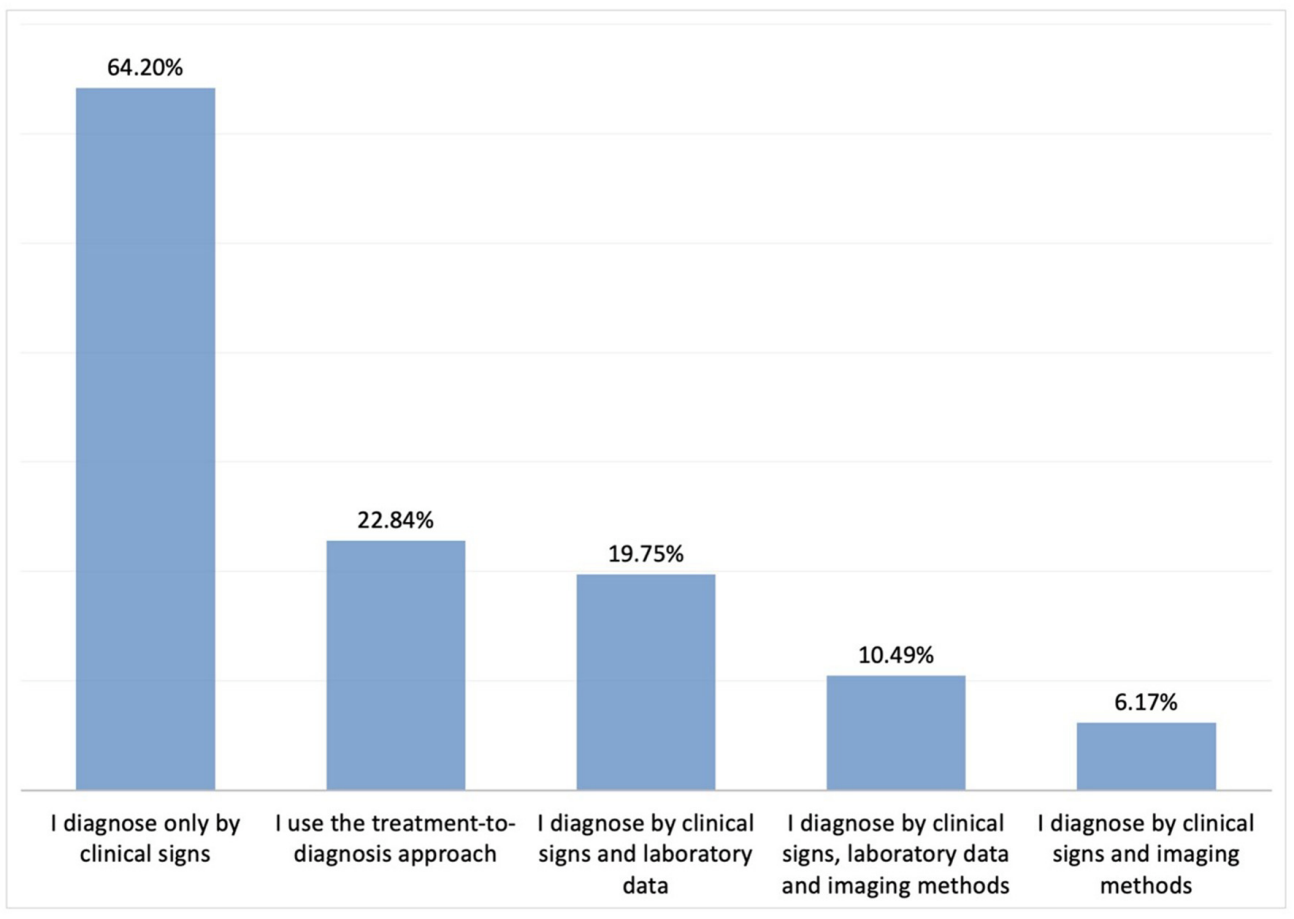
Additional investigations in the diagnosis of IC.

While the vast majority of participants stated that they mostly diagnosed IC based on clinical experience without the use of certain criteria, others reported utilising clinical experience in conjunction with the modified Wessel criteria (Figure 3). Again, the majority of the paediatricians reported not using any laboratory tests or imaging methods in diagnosing IC (Figure 4). The most common diagnostic test ordered by physicians was reportedly urinalysis (55.5%).

### Therapeutic Choices in Infantile Colic

#### General Therapeutic Choices and Relevant Rationales

**Figure 5:**
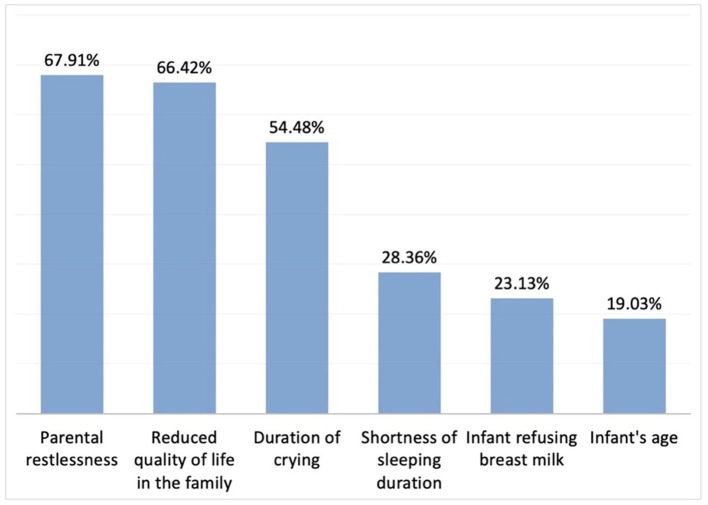
Considerations to initiate treatment in IC (*n* = 268). Here we provided the rest of the percentages of the chosen other answers: Infant being the first child; 3.73%, Growth retardation; 2.99% respectively.

Overall, 85.8% of the participants (*n* = 324) reported using a therapeutic approach in the patients they diagnose with IC. The 3 most important considerations for treatment initiation included parental restlessness, reduced quality of life in the family, and the duration of crying (Figure 5). As reported by the participants, the therapeutic choices were mostly based on previous experience, feedback, and scientific literature in 75.3, 62.3, and 38.4% of the cases, respectively (*n* = 268).

**Figure 6:**
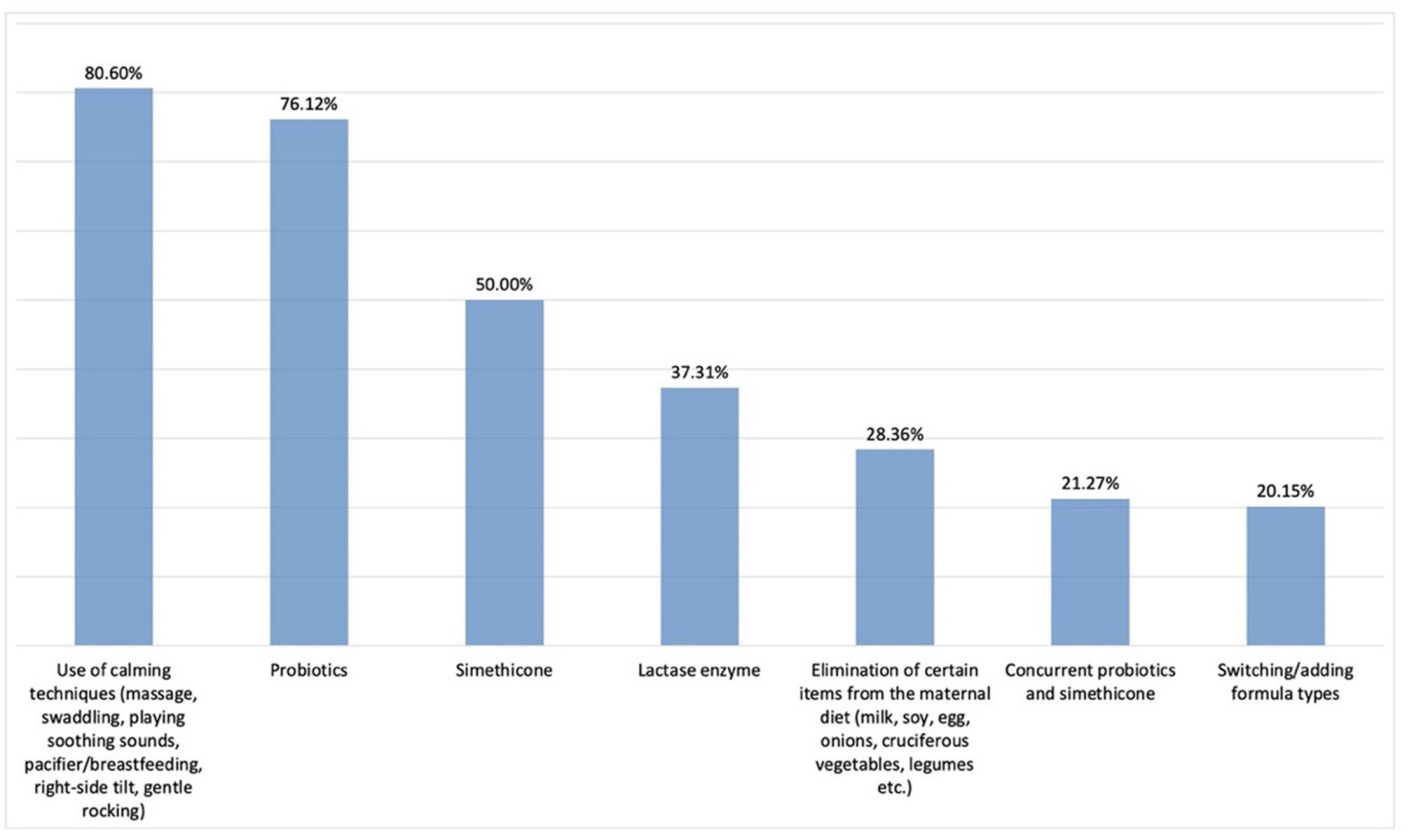
Therapeutic choices in IC (*n* = 268). Here we provided the rest of the percentages of the chosen other answers: Prebiotics; 16.79%, Addition of herbal tea to maternal diet; 13.81%, Symbiotics; 4.48%, Antispasmodics; 2.99%, Concurrent symbiotics and simethicone; 2.99%, Concurrent diet, antacids, and simethicone; 2.24%, Addition of herbal tea to infant's diet; 1.87%,Antacids (PPI);0.75%, Concurrent antacids and simethicone; 0.00% respectively.

According to the responses to this part of the questionnaire consisting of 16 items, the three most commonly utilised therapeutic options appeared to be calming techniques, probiotics, and simethicone (Figure 6).

#### Approaches According to Infant Age (Months)

Most paediatricians stated that their treatment choice would differ according to the age of the infant (54.5 vs. 45.5%, *n* = 268). A total of 139 participants responded to the questions about the use of different treatment modalities according to infant age in weeks and months. The participants provided information on their therapeutic choices in the following age groups: those up to 4 weeks of age (Group 1); between 4 weeks and 4 months of age (Group 2); and those older than 4 months of age (Group 3). The three most commonly preferred treatment options were probiotics, calming techniques, and simethicone in Group 1 (76.2, 61.1, and 30.9%, respectively); probiotics, calming techniques, and food elimination from mothers' diet in Group 2 (64.7, 60.4, and 43.1%, respectively); and consultation with gastroenterology specialists, probiotics, and food elimination from mothers' diet in Group 3 (45.3, 43.1, and 42.4%, respectively).

#### Approaches According to Infant Feeding Pattern

Infant feeding pattern was reported to have an impact on preferred treatments by 63.4% of the participants (*n* = 257). Among breast-fed infants, the most commonly preferred IC treatments in decreasing order of frequency were probiotics, calming techniques, simethicone (62.5, 56.5, 42.1%, respectively, for the 3 most common treatments; *n* = 152), while food elimination and using the lactase enzyme ranked in 4th and 5th, respectively. In infants fed with formula, adding or switching to another formula appeared to be the most commonly preferred treatment modality for IC (63.1%), followed by probiotics, calming techniques, simethicone, and use of lactase enzyme (62.5, 50, 35.5, and 23%, respectively; *n* = 152). Treatment modalities preferred in infants receiving breast milk+formula and/or additional food included probiotics in 1st place, followed by switching to/addition of another formula and calming techniques, both ranking 2nd at equal rates, simethicone ranking 3rd, food elimination ranking 4th, and use of lactase ranking 5th place (69, 50, 40, 34.8, and 29.6%, respectively; *n* = 152).

#### Assessment of Response to Treatment

**Figure 7:**
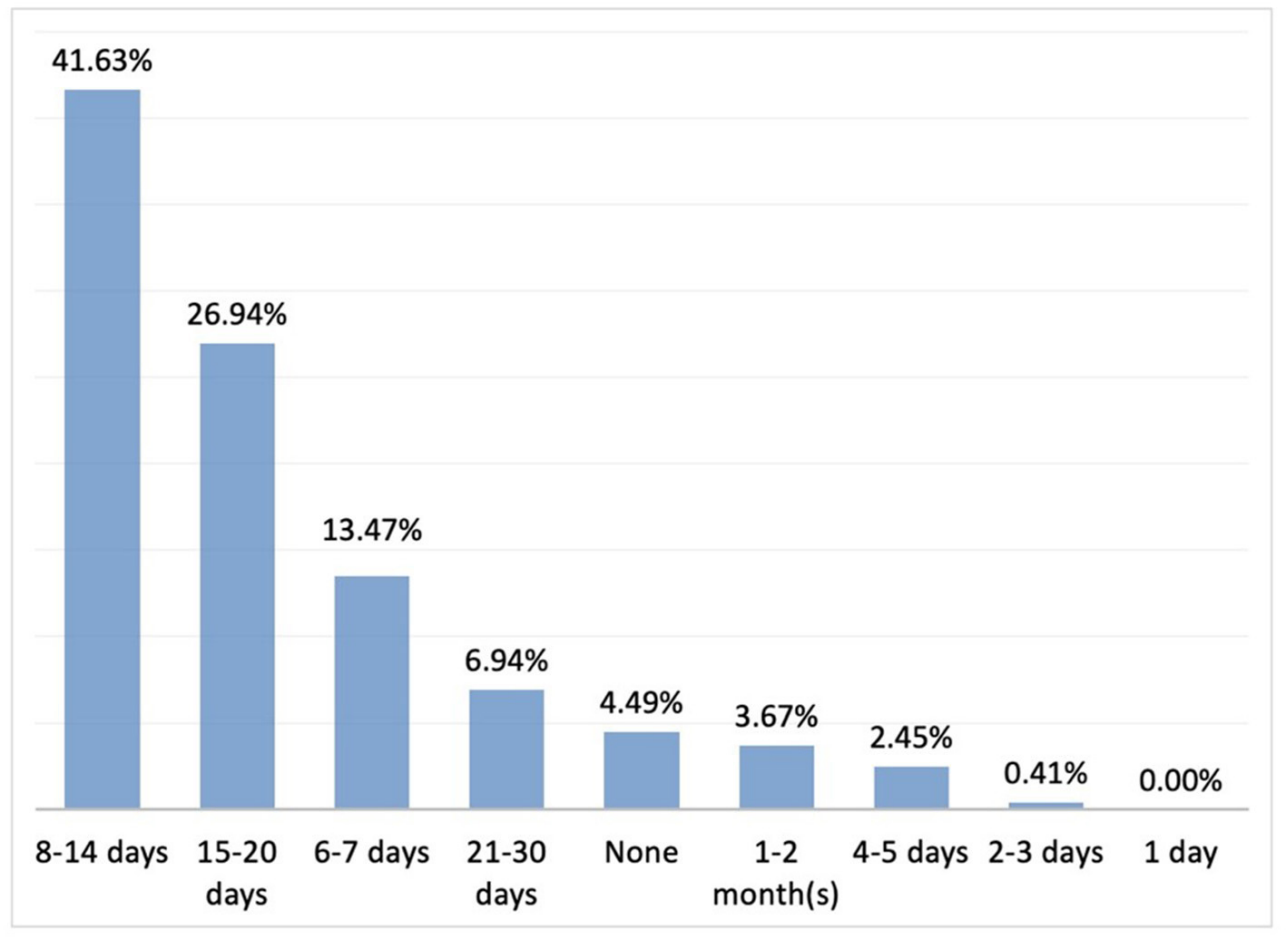
IC treatment switch (*n* = 245).

Most of the participants stated that they wait 8–14 days to observe the response to treatment and change the treatment (Figure 7).

The 3 parameters that were most commonly utilised to assess response to treatment included reduction in the duration of crying episodes, relaxation of the infant, and reduced restlessness of the parents (81.6, 74.6, and 62%, respectively; *n* = 245). These 3 clinical response parameters were reportedly followed by increased sleep duration, reduced crying episodes after feeding, relaxation in wind passing, improved feeding quality and duration, reduced number of defecations, and improved stool consistency (41.6, 32.6, 28.5, 25.7, and 5.3%, respectively; *n* = 245).

When improvement in sleep patterns and the rate of reduction in duration of crying were inquired to assess adequate response to IC treatment, the most commonly chosen items were observed to be improved sleep pattern by 50–79% and reduced duration of crying episodes by 25–49% (48.9 and 47.3%, respectively, *n* = 245). In case of treatment failure, most participants stated that they would proceed with workup to rule out organic pathologies or add a second treatment to the existing therapy (41.6 and 41.6%, respectively; *n* = 245). The 3rd most commonly preferred option was switching to another treatment (13%, *n* = 245).

### Preferred Treatments

#### Choice of Spasmolytic Agent

Of the 243 participants, only 32 (13.7%) reported a preference for spasmolytic agents for the treatment. The reasons cited for not preferring spasmolytic agents were the contraindication in infants under 6 months of age, assumed lack of efficacy in infantile colic, and avoidance of respiratory side effects (55.2, 42.7, and 18.2%, respectively; *n* = 208).

#### Choice of Probiotics

Overall, 97.9% of the participants reported using probiotics in the treatment of IC (*n* = 240). The most commonly preferred bacterial species were L. reuteri L. rhamnosus, B. lactis, L. paracasei and other species respectively (77.9, 59.7, 32.9, 4.3, and 1.7%; *n* = 231).

Although probiotics were the most commonly preferred treatment option for IC, some of the participants were found to utilise these in 2nd place or in different lines of treatment (63.64, 23.81, and 8.23%, respectively; *n* = 231). When inquired about in which patients they preferred probiotics, 68.4% of the participants reported this preference in those fed with formula and 50.2% in those fed with breast milk (*n* = 231).

While the majority of the participants preferred to use probiotics once a day, the most common two preferences as treatment duration were longer than 6 and 4 weeks respectively (89.1, 45.4, 32.4%, *n* = 231).

**Figure 8:**
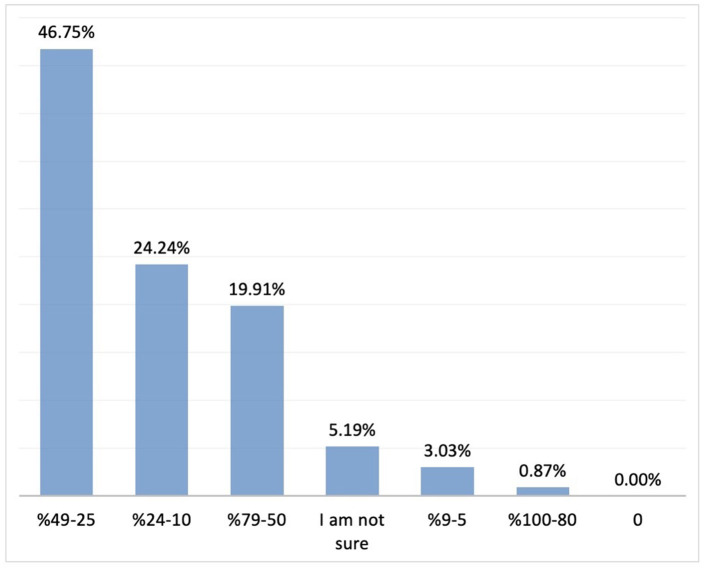
The rate of improvement in duration of crying with probiotics (*n* = 231). Y axis: response percentages, X axis: rate of improvement in sleep pattern percentages.

**Figure 9:**
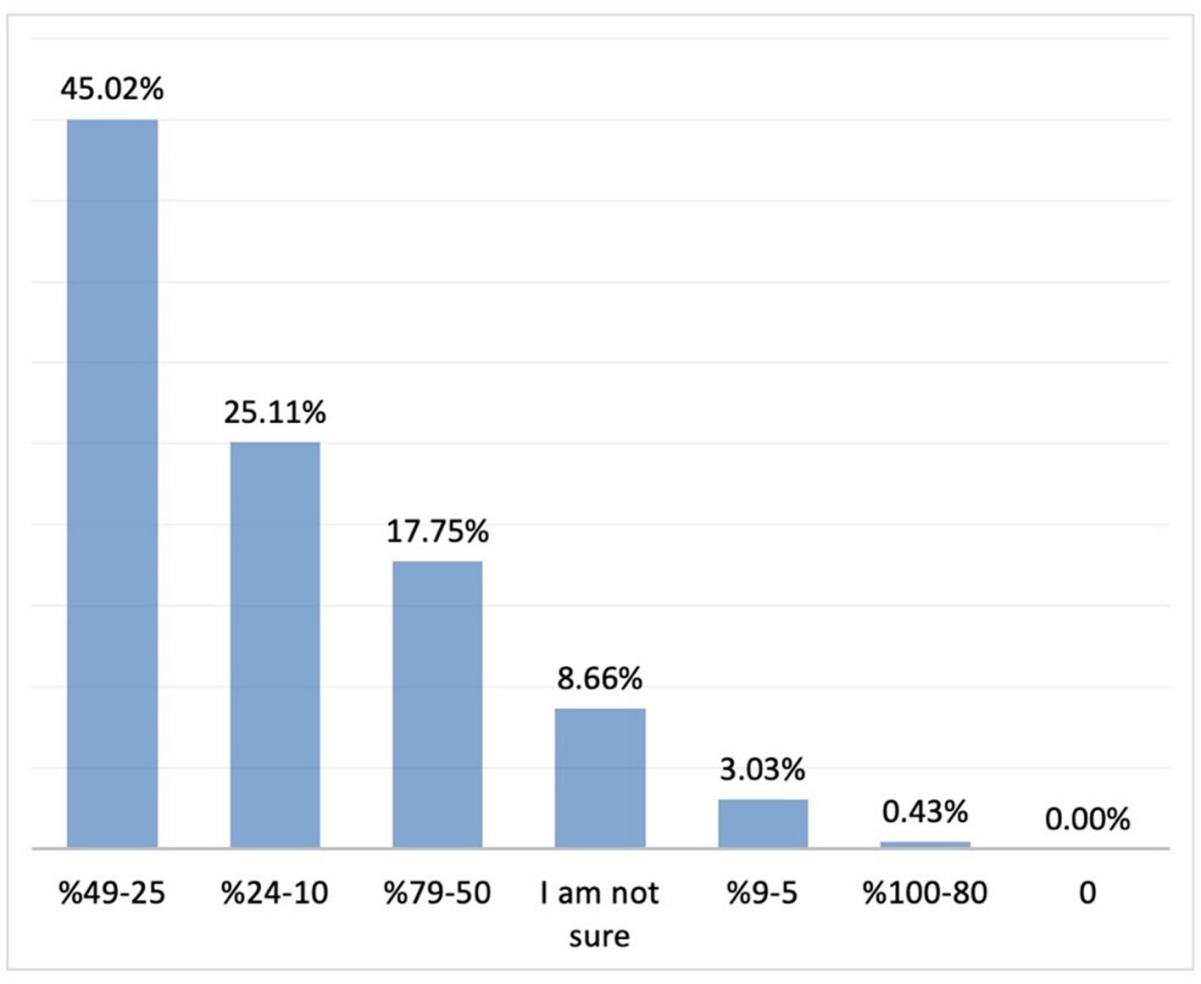
The rate of improvement in sleep pattern with probiotics (*n* = 231). Y axis: response percentages, X axis: rate of improvement in sleep pattern percentages.

The most commonly cited benefits of probiotics included reduced duration of crying episodes, facilitation of wind passing or defecation, relief of indigestion, and reduced crying episodes after feeding (70.5, 53.6, 48.9, and 44.5%, respectively; *n* = 231). Regarding participants' observations on improvement in duration of crying, 46.7% reported a 25–49% reduction in the duration of crying episodes, while 45% reported a 25–49% improvement in sleep pattern (*n* = 231) (Figures 8, 9).

#### Choice of Simethicone

Of the participants, 71.6% reported using simethicone for the treatment of IC (*n* = 236). Among IC treatments preferred, simethicone was generally prescribed within the first two lines of the treatment (1st line: 30.7%, 2nd line: 50.3%, *n* = 169) Simethicone was preferred in breastfed infants by 44.9% of the participants, and in formula-fed infants by 41.2% of the participants (*n* = 169).

Majority of the participants stated that they use simethicone treatment as 1–3 or 4–6 times daily (50.3 and 48.5%, respectively; *n* = 196). In most instances, 5–9 drops of simethicone was prescribed, followed in decreasing frequency by 10–14 drops, and 15 drops (56.2, 18.9, and 16.5%, respectively; *n* = 169).

Regarding the observations on the response to simethicone therapy, the most commonly cited response was “reduced duration of crying,” followed by reduced frequency of crying, facilitated passage of gas/stool, and prolongation of sleep (67.4, 47.3, 44.3, and 34.3%, respectively; *n* = 169). Most of the participants who preferred simethicone reported that it was effective for restlessness, with reduced parental restlessness and improved quality of life (84, 53.2, and 51.4%, respectively; *n* = 169).

**Figure 10:**
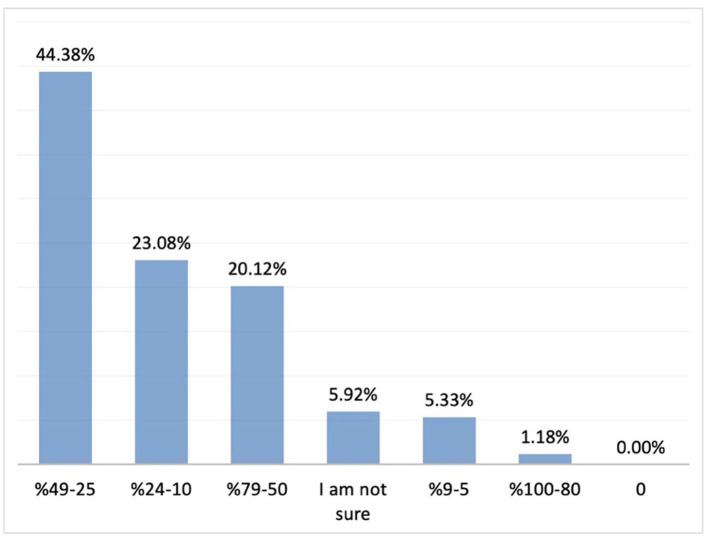
The rate of improvement in duration of crying with simethicone. Percent distribution; rate of improvement in the duration of crying.

**Figure 11:**
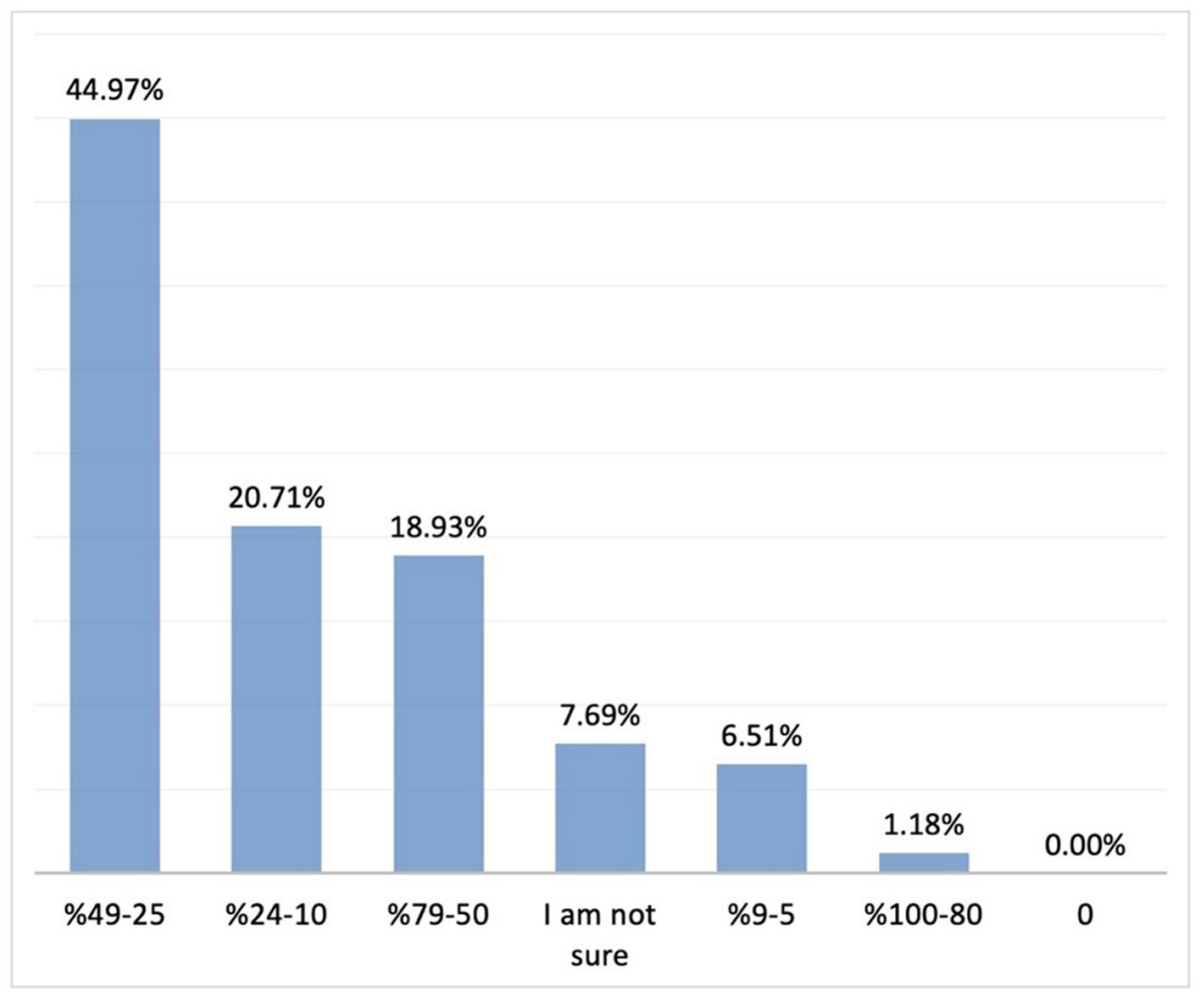
The rate of improvement in sleep pattern with simethicone.

A 25 to 49% reduction in the duration of crying episodes was reported by 44.38% of the participants, while 25 to 49% improvement in sleep pattern was reported by 44.97% (*n* = 169). (Figures 10, 11).

The most commonly cited reason for preferring simethicone was convenience of use, followed by absence of systemic absorption and rapid onset of action (43.7, 37.8, and 36.6%, respectively; *n* = 169). On the other hand, reasons cited for not preferring simethicone included perceived lack of improvement in clinical signs, reluctance about side effects, and inadequate clinical evidence (40.3%, 35.8%, and 26.8, respectively; *n* = 67).

## Discussion

The results of this survey showed that the diagnosis of infantile colic was mostly based on clinical experience alone among the participants and none of the participating physicians reported relying solely on Modified Wessel or Rome criteria apart from clinical experience. Available literature states that while Wessel's criteria for diagnosing infantile colic were abandoned, adopting the Rome IV criteria in clinical practise will help to reduce family distress by providing early and timely reassurance, education, and support to the parents of infants with colic (17).

Present survey study also found out that medical treatments were prescribed by the vast majority of the physicians and previous treatment outcome experience was the most important single determinant on their treatment preference. Similar to the present study, surveys conducted in Europe have also shown that the majority of physicians establish the diagnosis based on clinical experience alone (14, 15).

Population-based studies in Western countries have reported a mean prevalence 20% for IC (18). The lowest prevalence rates were reported from Denmark (6%) and Japan (2%) among infants up to 6 weeks of age, while the highest prevalence rates (17–47%) were reported from the UK in infants of 1–2 weeks of age (2). In a survey among German and Polish paediatricians, the proportion of infants diagnosed with IC was 41.6% for the German paediatricians, while this figure was 38% in another survey from Australia (14, 15). In the current study where all participants were paediatricians, IC diagnosis was most commonly reported in infants of 4–6 weeks of age, followed by those aged 2–4 weeks. Although most of the participating physicians consider their clinical experience sufficient for establishing a diagnosis of IC, the most commonly ordered laboratory test in those with persistent symptoms was urinalysis, similar to the practise of their Australian colleagues (15).

In a study evaluating the diagnosis and medical treatment of infantile colic in parents, the most common symptoms that led to a diagnosis of IC was crying for more than 3 h per day, and formula-fed infants were more likely to be diagnosed with IC as reported by the parents (16). Based on the participants' responses in the present study, the 5 most common symptoms leading to a diagnosis of IC were inconsolable crying, crying at the same time of the day, restlessness, parental restlessness, and sleep disturbance.

To date, the aetiology of infantile colic has not been fully elucidated and no associations have been described between birth weight, route of labour, and type of feeding. Potential risk factors include history of maternal smoking, advanced maternal age, and being the first baby, while more recently, increasing number of studies also suggest that dysbiosis, i.e. imbalance of the intestinal flora, may also play an etiological role. As in other multifactorial conditions with no clear-cut etiopathogenesis, there is currently no gold standard therapeutic approach for the management of IC treatment (19). In previous surveys conducted among physicians to investigate therapeutic choices in IC, 97 and 82% of German and Polish paediatricians, respectively, were found to opt for pharmaceutical treatment, while only 18% of Australian paediatricians preferred medical treatments (14, 15). In the current study, medical treatments were prescribed by 85.8% of the physicians. The first 3 factors that had the greatest effect on initiating treatment were parental restlessness, reduced quality of life in the family, and duration of crying episodes, while the single most important determinant of the physicians' choice of therapy appeared to be previous treatment outcome experience. Infant's age and type of feeding were found to affect the treatment choices of physicians. As in other studies, the most important factors leading physicians to treat infantile colic were observed to be restlessness or depression of the mother and high levels of anxiety in family members. Considering all these factors, it was determined that the physicians participating in this study mostly preferred the calming technique, which is a behavioural therapy method. This was followed by the preferred use of probiotics and simethicone. Similar to the present study, in a survey study conducted with German and Polish paediatricians to investigate the treatment approaches in IC, the first 3 options were pro/symbiotic, simethicone, and behavioural therapy, respectively (14). Among a group of Australian paediatricians, calming techniques, elimination diets, switch to hypoallergic infant formula, and medical treatment were used in 65%, 26%, 24%, and 18% of the IC cases, respectively (15).

Probiotics that are utilised most commonly in IC include Lactobacillus spp., Bifidobacterium spp., and particularly L. reuteri (20). The majority of participating in this study reported a preference for L. reuteri among probiotics, consistent with the literature. In a randomised controlled trial (RCT) involving 589 healthy full-term infants either fed with breast milk or infant formulas, L. reuteri DSM 17938 was found to prevent IC in both groups (21). In a meta-analysis of 7 randomised controlled studies, daily use of L. reuteri DSM 17938 (1 × 10^8^ colony forming units) for 21 or 28 days was associated with a 50- ]mins reduction in the duration of crying episodes (20). At odds with these positive results, the largest randomised controlled trial of probiotic intervention in infants with colic to date showed that L. reuteri DSM 17938 did not benefit a community sample of breast-fed infants and formula-fed infants with colic, and as a result, the researchers did not support a general recommendation for the use of probiotics to treat colic in infants (22). Additionally, many systematic reviews and metanalysis stated that there is no clear evidence that probiotics are more effective than placebo at preventing infantile colic; however, daily crying time appeared to reduce with probiotic use compared to placebo (23–25).

In contrast with short-term use of probiotics in most other studies, almost half of the participating physicians in the current study reported use of probiotics for more than 6 weeks Again, consistent with the literature, a 25–49% reduction in the duration of crying episodes was reported by the physicians who participated in the present study. Probiotics currently available on the market include food products or dietary supplements. To date, no probiotic products have been approved by the FDA or the Turkish Ministry of Health to treat, lessen, cure or prevent specific diseases. Furthermore, how well a probiotic works may differ from brand to brand and even from batch to batch within the same brand (26).

In the treatment of IC, controversial results have been observed regarding the use of simethicone, which reduces the surface tension of the intestinal mucosa, promotes the merging of intestinal bubbles, and facilitates passing of gas from intestines (27). A RCT by Sethi et al. reported fewer cases of crying episodes following administration of simethicone compared to placebo in infants with colic (28). In 2 other RCTs, although simethicone was associated with some general improvements, no difference compared placebo was observed in terms of IC symptoms (29, 30). However, these studies also differ with regard to definitions of infantile colic, inclusion criteria, and the criteria to determine response to treatment. On the other hand, in other studies involving the use of questionnaires among parents or physicians, both groups generally agreed on a symptomatic improvement through simethicone use in IC treatment. For instance, in a survey involving 4,004 parents, simethicone use was associated with significant reductions in the intensity of crying episodes as well as in symptoms of restlessness (16). In a study involving German and Polish paediatricians, 70% of the former group reported combined use of probiotics and simethicone, with 9% preferring an intensive regimen for simethicone, while the among the latter, only 34% reported combined use, with 49% utilising probiotics only, and 17% simethicone only (14). In the present study, 71.6% of the respondents reported a preference for simethicone use. However, in contrast with other reports in the literature, only 26% of the participating physicians reported combination of simethicone with other treatments.

Although simethicone should be administered at a dose of 15 drops every 4–6 h in infants, half of the paediatricians in our study reported recommending 5–9 drops to be administered 1–3 times per day (31). This may be associated with the presence of discrepant recommendations in online resources, other than the formal prescribing information. The respondent physicians reported a 25–49% improvement in the duration of crying episodes. Again, 84% of the participants reported positive effects of simethicone on restlessness, which was cited as the single most important factor for initiating medical treatment.

One limitation of the present study is the fact that the number of participants in this survey consisting mostly of Turkish paediatricians may be inadequate to represent the general population of paediatricians in Turkey. However, a closer look at the distribution of participants according to cities and different healthcare settings indicate a nearly homogeneous profile, likely to alleviate the effect of the above-mentioned limitation. We believe this study is important in that it is the first and most comprehensive survey in the current literature that investigates Paediatricians' diagnostic and therapeutic approaches in IC together with their perceptions of response to treatment. Future studies will have to focus on investigating the sufficiency of diagnostic criteria efficacy of these commonly used treatments by head-to-head trials. Evidence-based advice on the management of infantile colic will guide paediatricians and families through this stressful period.

In conclusion, this sample of Turkish paediatricians has shown that they mostly rely on clinical signs and symptoms, while mostly preferring urinalysis for patients deemed to require differentiating diagnosis with further laboratory workup. In the absence of a clear consensus regarding the treatment, the use of calming techniques is seen as one of the most important therapeutic choice by the participant paediatricians, probiotics appear to be the most commonly utilised type of supplement, while simethicone is reported to be the most commonly preferred medical treatment, with significant benefits reported for both.

## Data Availability Statement

The raw data supporting the conclusions of this article will be made available by the authors, without undue reservation.

## Author Contributions

SH, DC, IK, EÖ, TT, and HÖ contributed to development of survey questions, literature review, data analysis, and manuscript development. All authors contributed to the article and approved the submitted version.

## Funding

The authors declare that this study received funding from Pfizer. The funder was not involved in the study design, collection, analysis, interpretation of data, the writing of this article or the decision to submit it for publication. Medical writing and editorial support were provided by Remedium Consulting Group and was funded by Pfizer.

## Conflict of Interest

SH has acted in study advisory board of Pfizer. DC and TT have acted in advisory board of Pfizer. IK and EÖ have acted in advisory board of Pfizer and have received speaker honorarium from Pfizer. HÖ has acted in advisory board of Pfizer, and has received speaker honorarium from Pfizer and Chiesi Farmaceutici Turkey.

## Publisher's Note

All claims expressed in this article are solely those of the authors and do not necessarily represent those of their affiliated organizations, or those of the publisher, the editors and the reviewers. Any product that may be evaluated in this article, or claim that may be made by its manufacturer, is not guaranteed or endorsed by the publisher.
