# Supplementary material for: Diagnosis and Treatment Approaches in Infantile Colic (IC): Results of a Survey Among Paediatricians in Turkey
Source: Front Pediatr. 2021 Dec 23;9:779997. doi: 10.3389/fped.2021.779997 (PMC8734615; doi:10.3389/fped.2021.779997)
Supplement: Supplementary file 1 [file Data_Sheet_1.DOCX]

Supplementary Material

# Survey Questions: Diagnosis and Treatment Approaches in Infantile Colic Results of a Survey Among Pediatricians in Turkey

* 1. Are you a pediatrician?

☐ Yes

☐ No

* 2. Please specify the city you work in.

* 3. Please tick your gender.

☐ Female

☐ Male

* 4. How long have you been working as a pediatrician?

☐1-3 years

☐4-6 years

☐7-10 years

☐10 years and above

* 5. Please specify if you have a subspecialty.

* 6. Please specify the structure of the healthcare provider you work for (you may tick more than one option).

☐ Public Hospital

☐ Training and Research Hospital

☐ University Hospital

☐ City Hospital

☐ Private Hospital

☐ Doctor's Office

* 7. What is the average number of patients under 6 months of age you examine daily?

☐Less than 5

☐5-10

☐11-20

☐21-40

☐More than 40

* 8. Please specify the average number of patients you consider/diagnose with infantile colic (IC) among patients under 6 months of age you examine in a week.

☐ 1-4

☐ 5-10

☐ 11-15

☐ 16-20

☐>20

* 9. Please choose the symptoms/signs that most suggest a diagnosis of IC to you (maximum 5 options).

☐ Inability to gain weight, delay in growth percentile

☐ Stool consistency (loose/watery), change in odor

☐ Mucus in stool

☐ Restlessness

☐ Vomiting after meal

☐ Uncontrollable crying

☐ Crying at the same time every day

☐ Increase in crying duration

☐ Sleep disturbance

☐ Eczema

☐ Parental restlessness

☐ Bursting gas, burping

☐ Tendency to stop breast milk consumption

* 10. Please choose the familial factor or factors that you think are most associated with IC (maximum 5 options).

☐ Advanced maternal age

☐ Smoker mother

☐ Being the first child

☐ High parental anxiety

☐ Inadequate infant-parent (caregiver) relationship

☐ Mother's educational status

☐ Socioeconomic status

☐ Parental conflict

☐ Being a nuclear family

☐ Working mother

☐ None

* 11. When diagnosing IC, I mostly

☐ use my clinical experience + Wessel's criteria.

☐ use my clinical experience + Rome IV criteria.

☐ use my clinical experience + modified Wessel's criteria.

☐ use my clinical experience + review all 3 criteria.

☐ evaluate with my clinical experience only.

☐ None

* 12. I mostly diagnose IC............... (if one of the choices is the last option, you may make 2 choices)

☐ based on clinical signs only.

☐ based on clinical signs and laboratory findings.

☐ based on clinical signs and imaging techniques.

☐ based on clinical signs, laboratory findings, and imaging techniques.

☐ by going from treatment to diagnosis.

* 13. If you use laboratory testing for the diagnosis of IC, which one(s) do you mostly order? (If your choices are not the last 2 options, you may tick more than one option)

☐ Blood test

☐ Urine test

☐ Stool test

☐ Tests for the detection of food allergy

☐ All

☐ None

* 14. In which weeks do you most frequently diagnose IC?

☐ First 2 weeks

☐ 2-4 weeks

☐ 4-6 weeks

☐ >6 weeks

* 15. Do you recommend treatment to your patients you diagnose with IC?

☐ Yes

☐ No

* 16. Please choose the factors that most influence your decision to initiate treatment for IC patients (maximum 3 options).

☐ Infant's age

☐ Crying duration

☐ Developmental delay

☐ Short sleep duration

☐ Refusal to take breast milk

☐ Parental restlessness

☐ Decreased quality of life of the family

☐ Being the first child

* 17. Please tick the treatment modality or modalities you use most frequently for your patients you diagnose with IC (maximum 5 options).

☐ Antacid (PPI)

☐ Antispasmodic

☐ Probiotic

☐ Simethicone

☐ Lactase enzyme

☐ Prebiotic

☐ Symbiotic

☐ Antacid and simethicone simultaneously

☐ Symbiotic and simethicone simultaneously

☐ Diet, antacid, and simethicone simultaneously

☐ Simethicone and probiotic simultaneously

☐ Change or addition of formula types

☐ Use of calming methods (massage, swaddling, having the infant listen to Shhh sounds, pacifier/breastfeeding, lying on the right side, gentle swaying)

☐ Elimination of certain substances from the mother's diet (milk, soy, eggs, onions, crucifers, legumes, etc.)

☐ Addition of herbal tea to the mother's diet

☐ Addition of herbal tea to the baby's diet

* 18. Please tick the factor or factors that are most effective in making your treatment choice (maximum 3 options)

☐ My previous experiences

☐ Feedbacks I have received

☐ Studies on the subject

☐ Guideline recommendations

☐ High level of anxiety of the family

☐ Developmental delay due to malnutrition

☐ Severity of symptoms

* 19. Does your treatment choice change depending on the infant's age?

☐ Yes

☐ No

* 20. Please tick the treatment modalities you most frequently prefer for infants aged up to 4 weeks (maximum 5 options)

☐ Antacid (PPI)

☐ Antispasmodic

☐ Probiotic

☐ Simethicone

☐ Lactase enzyme

☐ Prebiotic

☐ Symbiotic

☐ Antacid and simethicone simultaneously

☐ Symbiotic and simethicone simultaneously

☐ Diet, antacid, and simethicone simultaneously

☐ Simethicone and probiotic simultaneously

☐ Change or addition of formula types

☐ Use of calming methods

☐ Elimination of certain substances from the mother's diet

☐ Addition of herbal tea to the mother's diet

☐ Addition of herbal tea to the infant’s diet

* 21. Please tick the treatment modalities you most frequently prefer for infants aged 4 weeks to 4 months (maximum 5 options)

☐ Antacid (PPI)

☐ Antispasmodic

☐ Probiotic

☐ Simethicone

☐ Lactase enzyme

☐ Prebiotic

☐ Symbiotic

☐ Antacid and simethicone simultaneously

☐ Symbiotic and simethicone simultaneously

☐ Diet, antacid, and simethicone simultaneously

☐ Simethicone and probiotic simultaneously

☐ Change or addition of formula types

☐ Use of calming methods

☐ Elimination of certain substances from the mother's diet

☐ Addition of herbal tea to the mother's diet

☐ Addition of herbal tea to the infant’s diet

☐Arrangement of treatment by gastroenterology consultation

*22. Please tick the treatment modalities you most frequently prefer for infants aged 4 months and older (maximum 5 options)

☐ Antacid (PPI)

☐ Antispasmodic

☐ Probiotic

☐ Simethicone

☐ Lactase enzyme

☐ Prebiotic

☐ Symbiotic

☐ Antacid and simethicone simultaneously

☐ Symbiotic and simethicone simultaneously

☐ Diet, antacid, and simethicone simultaneously

☐ Simethicone and probiotic simultaneously

☐ Change or addition of formula types

☐ Use of calming methods

☐ Elimination of certain substances from the mother's diet

☐ Addition of herbal tea to the mother's diet

☐ Addition of herbal tea to the infant’s diet

☐ Arrangement of treatment by gastroenterology consultation

*23. Does your treatment choice change depending on the infant's diet (fed with breast milk / formula / breast milk + formula)?

☐ Yes

☐ No

* 24. Please tick the treatment modalities you most frequently prefer for infants fed with breast milk (maximum 5 options)

☐ Antacid (PPI)

☐ Antispasmodic

☐ Probiotic

☐ Simethicone

☐ Lactase enzyme

☐ Prebiotic

☐ Symbiotic

☐ Antacid and simethicone simultaneously

☐ Symbiotic and simethicone simultaneously

☐ Diet, antacid, and simethicone simultaneously

☐ Simethicone and probiotic simultaneously

☐ Change or addition of formula types

☐ Use of calming methods

☐ Elimination of certain substances from the mother's diet

☐ Addition of herbal tea to the mother's diet

☐ Addition of herbal tea to the infant’s diet

* 25. Please tick the treatment modalities you most frequently prefer for infants fed with formula (maximum 5 options)

☐ Antacid (PPI)

☐ Antispasmodic

☐ Probiotic

☐ Simethicone

☐ Lactase enzyme

☐ Prebiotic

☐ Symbiotic

☐ Antacid and simethicone simultaneously

☐ Symbiotic and simethicone simultaneously

☐ Diet, antacid, and simethicone simultaneously

☐ Simethicone and probiotic simultaneously

☐ Change or addition of formula types

☐ Use of calming methods

☐ Elimination of certain substances from the mother's diet

☐ Addition of herbal tea to the mother's diet

☐ Addition of herbal tea to the infant’s diet

* 26. Please tick the treatment modalities you most frequently prefer for infants fed with breast milk + formula and/or supplementary food (maximum 5 options)

☐ Antacid (PPI)

☐ Antispasmodic

☐ Probiotic

☐ Simethicone

☐ Lactase enzyme

☐ Prebiotic

☐ Symbiotic

☐ Antacid and simethicone simultaneously

☐ Symbiotic and simethicone simultaneously

☐ Diet, antacid, and simethicone simultaneously

☐ Simethicone and probiotic simultaneously

☐ Change or addition of formula types

☐ Use of calming methods

☐ Elimination of certain substances from the mother's diet

☐ Addition of herbal tea to the mother's diet

☐ Addition of herbal tea to the infant’s diet

*27. When do you mostly re-evaluate your patients to question treatment response in the treatment of IC?

☐ 1 day later

☐ 2-3 days later

☐ 4-5 days later

☐ 6-7 days later

☐ 8-14 days later

☐ After the 14th day

☐ None

* 28. How long do you wait on average before switching from one treatment option to another in the treatment of IC??

☐ 1 day

☐ 2-3 days

☐ 4-5 days

☐ 6-7 days

☐ 8-14 days

☐ 15-20 days

☐ 21-30 days

☐ 1-2 months

☐ None

* 29. What are the clinical response parameters you use to evaluate the treatment response in the treatment of IC? (you may choose more than one option)

☐ Increase in sleep duration

☐ Reduction in crying duration

☐ Improvement in nutritional quality and duration

☐ Reduction in crying periods after feeding

☐ Reduction in the number of defecations and improvement in form

☐ Relief of passing gas

☐ Infant's peace

☐ Reduction in parental restlessness

☐ All

☐ None

* 30. How much of an improvement would you expect to see in sleep routine/pattern to say that adequate treatment response has been achieved in the treatment of IC?

☐ 100-80%

☐ 79-50%

☐ 49-25%

☐ 24-10%

☐ 9-5%

*31. How much reduction would you expect in crying duration to say that adequate treatment response has been achieved in the treatment of IC?

☐ 100-80%

☐ 79-50%

☐ 49-25%

☐ 24-10%

☐ 9-5%

*32. What would your second approach be if you think that the initial treatment you gave was not enough in the treatment of IC?

☐ I switch to another treatment.

☐ I add a second form of treatment to the current treatment.

☐ I make multiple additions to the current treatment.

☐ I re-evaluate the patient to rule out organic pathologies.

☐ I consult the gastroenterology department.

☐ None

* 33. Do you use antispasmodic drugs for the treatment of IC?

☐ Yes

☐ No

*34. If you do not use antispasmodic drugs for the treatment of IC, what is the reason? (you may tick more than one option)

☐ Because they are contraindicated in children under 6 months of age.

☐ Because I refrain from respiratory side effects.

☐ Because I think they do not reduce the symptoms of infantile colic.

☐ None

* 35. Do you use probiotics for the treatment of IC??

☐ Yes

☐ No

* 36. If you use probiotics for the treatment of IC, which types of bacteria do you prefer? (you may tick more than one option)

☐ L. Reuteri

☐ B. Lactis

☐ L. Rhamnosus

☐ L. Paracasei

☐ Other:

* 37. In which line do you generally prefer probiotic treatment for the treatment of IC?

☐ 1st Line

☐ 2nd Line

☐ 3rd Line

☐ 4th Line

☐ 5th Line

☐ Last line

☐ I prefer it in variable lines.

* 38. In which cases/conditions do you prefer probiotic treatment for IC? (you may tick more than one option)

☐ I prefer it for those who are breastfed.

☐ I prefer it for those who are fed with formula.

☐ I prefer it for those whose number or form of defecations is not normal.

☐ I do not prefer it alone, but I definitely add it to the combination treatment.

☐ I give it only to have tried another agent when I am desperate.

☐ I give it to reduce the anxiety of parents.

* 39. How many times a day do you recommend using probiotics?

☐ Once

☐ 1-3 times

☐ 4-6 times

☐ None

* 40. For how long do you recommend using probiotics?

☐ 1 week

☐ 2 weeks

☐ 3 weeks

☐ 4 weeks

☐ 5 weeks

☐ 6 weeks

☐ Longer than 6 weeks

☐ None

* 41. What are your observations of response to probiotic treatment based on experience? (you may tick more than one option)

☐ Shortens the infant's crying duration

☐ Prolongs the infant's sleep duration

☐ Solves the infant's digestive problems.

☐ Prolongs the infant's feeding (sucking) duration

☐ Reduces crying periods after feeding

☐ Contributes to weight gain

☐ Facilitates passing gas/defecation

☐ None

*42. For which conditions that may be caused by IC do you think probiotic treatment is beneficial? (you may tick more than one option)

☐ Inability to gain weight or delay in growth percentile

☐ Change in defecation frequency and stool consistency

☐ Mucus in stool

☐ Restlessness

☐ Vomiting after meals (feeding)

☐ Tendency to stop breast milk consumption

☐ Improving the quality of life

☐ None

* 43. I think probiotics reduce crying duration by............... in IC.

☐ 100-80%

☐ 79-50%

☐ 49-25%

☐ 24-10%

☐ 9-5%

☐ 0%

☐ Not sure.

* 44. I think probiotics improve sleep routine/pattern by .................. in IC.

☐ 100-80%

☐ 79-50%

☐ 49-25%

☐ 24-10%

☐ 9-5%

☐ 0%

☐ Not sure.

* 45. What are your reasons for not preferring probiotics for IC? (you may tick more than one option)

☐ I do not think they improve clinical signs.

☐ I refrain from side effects

☐ I do not find the clinical study results sufficient

☐ I do not consider it appropriate to give medical treatment to infants under 6 months of age.

☐ None

* 46. Do you use simethicone for the treatment of IC?

☐ Yes

☐ No

* 47. In which line do you generally choose simethicone for IC??

☐ 1st Line

☐ 2nd Line

☐ 3rd Line

☐ 4th Line

☐ 5th Line

☐ Last line

☐ I prefer it in variable lines.

* 48. In which cases do you prefer simethicone treatment for IC? (you may tick more than one option)

☐ I prefer it for those who are breastfed.

☐ I prefer it for those who are fed with formula.

☐ I prefer it for those whose number or form of defecations is not normal.

☐ I do not prefer it alone, but I definitely add it to the combination treatment.

☐ I give it only to have tried another agent when I am desperate.

☐ I give it to reduce the anxiety of parents.

*49. How many times a day do you recommend using simethicone?

☐ 1-3 times

☐ 4-6 times

☐ 7-9 times

☐ 10-12 times

☐ None

* 50. How many drops of simethicone do you recommend using each time?

☐ More than 15 drops

☐ 15 drops

☐ 10-14 drops

☐ 5-9 drops

☐ 1-4 drops

☐ None

* 51. What are your observations of response to simethicone treatment considering your clinical experience? (you may tick more than one option)

☐ Shortens the infant's crying duration

☐ Prolongs the infant's sleep duration

☐ Solves the infant's digestive problems.

☐ Prolongs the infant's feeding (sucking) duration

☐ Reduces crying periods after feeding

☐ Contributes to weight gain

☐ Facilitates passing gas/defecation

☐ None

* 52. Do you have any reasons for preferring simethicone treatment based on the properties of the drug, and if so, what are they? (you may tick more than one option)

☐ Rapid onset of action

☐ Absence of systemic absorption

☐ Long duration of efficacy

☐ Ease of use

☐ I do not have a preference based on the properties of the drug.

* 53. For which conditions that may be caused by IC do you think simethicone treatment is beneficial? (you may tick more than one option)

☐ Inability to gain weight or delay in growth percentile

☐ Change in defecation frequency and stool consistency

☐ Mucus in stool

☐ Restlessness

☐ Vomiting after meals (feeding)

☐ Tendency to stop breast milk consumption

☐ Improving the quality of life

☐ None

* 54. I think simethicone reduces crying time by............... in IC.

☐ 100-80%

☐ 79-50%

☐ 49-25%

☐ 24-10%

☐ 9-5%

☐ 0%

☐ Not sure.

* 55. I think simethicone improves sleep routine by .................. in IC.

☐ 100-80%

☐ 79-50%

☐ 49-25%

☐ 24-10%

☐ 9-5%

☐ 0%

☐ Not sure.

* 56. What are your reasons for not preferring simethicone treatment for IC? (you may tick more than one option)

☐ I do not think it improves clinical signs.

☐ I refrain from side effects

☐ I do not find the clinical study results sufficient

☐ I do not consider it appropriate to give medical treatment to infants under 6 months of age.

☐ None
